# Interactions between prostaglandins, leukotrienes and HIV-1: Possible implications for the central nervous system

**DOI:** 10.1186/1742-4690-9-4

**Published:** 2012-01-11

**Authors:** Jonathan Bertin, Corinne Barat, Sylvie Méthot, Michel J Tremblay

**Affiliations:** 1 Centre de Recherche en Infectiologie, Centre Hospitalier Universitaire de Québec - CHUL, 2705 boul. Laurier, Québec (QC), Canada, G1V 4G2; 2 Département de Microbiologie-Infectiologie et Immunologie, Faculté de médecine, Université Laval, Québec, Canada

**Keywords:** HIV-1, Eicosanoids, Prostaglandins, Leukotrienes, NS-398, COX-2, 5-LO, Central Nervous System

## Abstract

In HIV-1-infected individuals, there is often discordance between viremia in peripheral blood and viral load found in the central nervous system (CNS). Although the viral burden is often lower in the CNS compartment than in the plasma, neuroinflammation is present in most infected individuals, albeit attenuated by the current combined antiretroviral therapy. The HIV-1-associated neurological complications are thought to result not only from direct viral replication, but also from the subsequent neuroinflammatory processes. The eicosanoids - prostanoids and leukotrienes - are known as potent inflammatory lipid mediators. They are often present in neuroinflammatory diseases, notably HIV-1 infection. Their exact modulatory role in HIV-1 infection is, however, still poorly understood, especially in the CNS compartment. Nonetheless, a handful of studies have provided evidence as to how these lipid mediators can modulate HIV-1 infection. This review summarizes findings indicating how eicosanoids may influence the progression of neuroAIDS.

## 1. Background

HIV-1, which will be referred to as HIV throughout this review, is widely known to cause devastating effects on the immune system. Another important target is the central nervous system (CNS), where macrophages and microglial cells are the cell types predominantly infected by HIV and responsible for virus propagation within the CNS. The effect of various molecules that act as activators or suppressors in the modulation of HIV replication dictates the viral load in the CNS. Although several such molecules have been shown to be involved in the neuropathogenesis of HIV infection and in the development of HIV-associated dementia (HAD), the relative importance of several others remains elusive. Of these, eicosanoids - more precisely prostaglandins (PGs) and leukotrienes (LTs) - have gained some attention during recent years for their implication in HIV pathogenesis. It is well documented that these lipid mediators perform multiple functions in many biological systems, mainly in inflammation and immunity. In the CNS, they play important physiological roles, notably in the resolution of inflammation and neuroprotective bioactivity. However, little is yet known on the complex relationships between HIV and these soluble mediators in the CNS. The current review will thus focus on the putative contribution of eicosanoid derivatives, namely PGs and LTs, to viral load and HIV-mediated neurological complications.

## 2. HIV and the CNS

### NeuroAIDS

It is now well accepted that HIV can cross the blood-brain barrier (BBB) and infect the CNS early, during the acute phase of the infection [1]. In the late stages of the infection, a wide range of neuropathologic complications are observed, such as neurotoxicity, neurodegeneration, HIV encephalitis and associated neurocognitive deficits. Neurological disorders are still strikingly prevalent, reaching up to 50% of virus-infected individuals during the course of the disease [2]. Clinical manifestations consist in a triad of neurological symptoms including cognitive, motor and behavioural impairments, better known as HAD. Before the introduction of the highly active antiviral therapy (HAART) in 1995, most of the infected population developed HAD at the late stage of the disease. HAART has reduced this rate drastically. However, a more subtle form of CNS dysfunction, known as minor cognitive motor disorder (MCMD) continues to prevail in HIV-carrying persons. Symptoms of MCMD include memory loss, as well as reduction of cognitive and computational functions [3]. Thus, the neurological manifestations and the inflammatory cascade underlying the infection of the CNS by HIV are far from being controlled by HAART. In fact, the relative CNS penetration-effectiveness (CPE) scores for different antiretroviral drugs used in HAART vary considerably, from very poor (e.g. enfuvirtide and nelfinavir) to high penetration (e.g. nevirapine and zidovudine) [4,5]. Nevertheless, it has been shown that the clinical outcome of CNS impairments related to HIV infection does not depend on the penetration efficacy of administered antiretroviral drugs [4]. In some cases, HIV-associated neurological disorders are actually worsened by the use of antiretrovirals [6]. A recent study has notably shown that maraviroc, a widely used CCR5 antagonist, actually increases microglia proinflammatory activation by inducing PGs synthesis, which may have the potential to exacerbate neurological complications [7].

Discordance between plasma and CSF (cerebrospinal fluid) viral loads in HIV-infected individuals has been documented in the literature [8-10]. Interestingly, HAART treatment seems to reduce the viral RNA concentrations more slowly in the CSF of some patients than in their plasma [11]. As a corollary, some studies have also shown that progression of HIV titers in the CNS occurs more slowly than in the plasma of patients with no neurological symptoms associated to HIV infection [12,13]. How the human body can control the viral load in the CNS is still not fully understood. Moreover, CNS viral strains may genetically differ over time from those found in the peripheral blood [14]. In the light of these observations, an involvement of cerebral innate immunity is probable. Indeed, it has been shown in non-human primate models that simian immunodeficiency virus (SIV) replication is more efficiently controlled in the CNS compartment than in the plasma [15]. In these models, the innate immune response seems to act as a key regulator of the viral burden in the CNS [16].

### Entry in and infection of the CNS by HIV

A wealth of knowledge has been published on the Trojan horse model describing how the virus may enter the CNS via infected leukocytes (mostly monocytes and/or CD4^+ ^T lymphocytes) or via cell-independent pathways [17-21]. This process is facilitated by a BBB disruption brought about by circulating microbial endotoxins - such as lipopolysaccharide found on Gram-negative bacteria - that enter the blood stream by translocation from the gastrointestinal tract during the acute phase of HIV infection [22-24] and by soluble HIV proteins such as Tat, Nef and gp120 [25]. HIV is then considered to be compartmentalized, on the one hand in the peripheral immune system, and, on the other hand, in the CNS. Once the virus enters the latter compartment, perivascular macrophages and microglia are the main targets for productive HIV infection. These cells can notably act as stable reservoirs for HIV latency (reviewed in [26]). Moreover, the infection and activation of these cells leading to production of proinflammatory mediators in the CNS underlie the pathogenesis of neuroAIDS [27]. Indeed, these inflammatory molecules do not only impact the function of surrounding cells, but can also alter tight junctions of the BBB, and enhance monocyte recruitment [28]. Among these secreted factors are eicosanoids, the focus of this paper.

### Microglia and HIV

Microglial cells are tissue macrophages that are ubiquitously distributed in the CNS. They serve as neuroprotective cells and promote neurotrophic functions [29]. It is now believed that microglia colonize the CNS from two pools of myeloid cells. The first progenitors that enter the embryonic and foetal CNS derive from hematopoietic cell precursors [30,31]. The second wave consists in monocytes, predominantly expressing some specific surface markers (i.e. CD11, CD14 and CD16), that enter and colonize the CNS during the postnatal period (infiltrating microglia) [32-34]. Microglial cells play many important roles in homeostasis. They can act as antigen-presenting cells, remove tissue debris following a brain trauma, cross-talk with astrocytes in order to regulate their proliferation, and produce cytokines and chemokines, as well as other soluble factors known to be involved in immunologic response, neuroinflammation and neurodegeneration [35-39].

As mentioned above, HIV once located within the CNS will predominantly target microglial cells and perivascular macrophages for productive infection. The virus-encoded external envelope glycoprotein gp120 utilizes different chemokine receptors and adhesion molecules in combination with CD4 to establish an initial contact with targets and eventually gains entry inside a host cell. It is well documented that microglia are mostly permissive to R5-tropic strains of HIV, which enter via the CCR5 coreceptor [40-42]. However, some data suggest that certain strains of HIV can also partly utilize CCR3, therefore suggesting some degree of complexity in the coreceptor usage by the virus on the surface of microglia [42,43].

HIV and certain virus-induced soluble factors have been shown to activate microglial cells. Interestingly, there is a better correlation between HAD and microglial activation than with the viral load [44]. The gp120 envelope protein is one of many viral components released in infected tissues. It has been shown to colocalize with microglia in the brain of HIV-infected patients [45]. Exposure of microglia to gp120 has been reported to induce an activation response, including the release of several molecules - such as TNF-α, IL-6, IL-1β, GM-CSF and reactive oxygen species - that induce CNS inflammation and neurotoxicity [19,46,47]. Another HIV protein, namely Tat, can also be found in the CSF and brain of HIV-infected individuals [48]. Treatment of human microglial cells with exogenous Tat can trigger the secretion of a broad range of proinflammatory molecules (e.g. MCP-1, IL-8, MIP-1α, MIP-1β and RANTES) [19].

Experiments aimed at defining the complex interactions between HIV and human microglia have used purified primary microglial cells obtained from human foetal brain tissues or brain specimens of patients undergoing surgery. However, the limited quantity of microglia that can be thus obtained has been a limiting step. Interestingly, a novel model of primary human microglia called human monocyte-derived microglia-like cells (MDMi) has been developed [49]. The phenotype of these cells resembles that of primary infiltrating microglia found in the CNS, and they can support productive HIV infection. This experimental model of human microglia has been recently proven to be useful for studying *in vitro *HIV infection in the CNS context [50]. Alternatively, animal models using SIV have been developed, which may prove useful for correlating the *in vitro *studies with an *in vivo *approach [51].

### Astrocytes and HIV

Astrocytes are the most abundant glial cells in the CNS. They play several important roles, such as regulating the external environment of neurons, participating in the physical structuring of the brain, providing metabolites to neurons, and maintaining the BBB integrity. Although perivascular macrophages and microglia are the cells that support productive virus infection in the CNS, astrocytes are considered by many to support a restricted virus replication. Indeed, only a subpopulation of productively infected astrocytes can be detected *in vivo *in HAD patients by sensitive techniques that detect virus nucleic acids [52-55], although an extensive infection of these cells has also been reported in demented infected individuals [56]. Nonetheless, much interest has lately been focused on the infectability of astrocytes. Their limited susceptibility to productive HIV infection has been attributed to a pH-independent restriction at the virus entry step [53,54] and an inhibition of the Rev protein by a host restriction factor called Risp [57].

Latent HIV infection of astrocytes results in considerable changes in the host gene expression pattern [58,59]. Consequently, the secretome of infected astrocytes has a major impact on neuronal cell death and BBB dysregulation, and it correlates with HIV neuropathogenesis and HAD severity [20,52]. Furthermore, recent findings suggest that the infection of a small number of astrocytes is sufficient to disrupt the BBB integrity by a gap junction-dependent mechanism [60], thus reinforcing the proposed important role for these few infected astrocytes in the HIV-induced CNS damage.

Interestingly, cross-talk between HIV-infected astrocytes and microglia has important impacts on neuroinflammation and neurodegeneration processes seen in the CNS. Indeed, HIV infection of astrocytes can increase the neurotoxicity of infected microglia and modulate viral growth and compensatory regulatory pathways in these cells [61]. Therefore, the very low number of virus-infected astrocytes can still play an important role in the development of HIV-associated encephalitis and neurological impairments.

## 3. Eicosanoids

Eicosanoids are signalling molecules that are generated through an oxidative pathway from polyunsaturated fatty acids, notably arachidonic acid (AA), an n-6 fatty acid (or omega-6), and eicosapentaenoic/docosahexaenoic, an n-3 fatty acid. The eicosanoids are considered as local hormones that include prostanoids (i.e. PGs, prostacyclins and thromboxanes), LTs, lipoxins, hepoxilins and hydroxyeicosatetraenoic (HETE) acid. They are important homeostatic or proinflammatory lipid mediators that exert autocrine and paracrine activities in diverse physiologic responses (e.g. vasomodulation, platelet aggregation and bronchodilatation). Of more relevance here, PGs and LTs strongly regulate our innate and humoral immunity responses. Production of several eicosanoids is considerably increased during inflammation, and they are involved in the pathogenesis of various diseases.

### Biosynthesis pathway from AA

**Figure 1:**
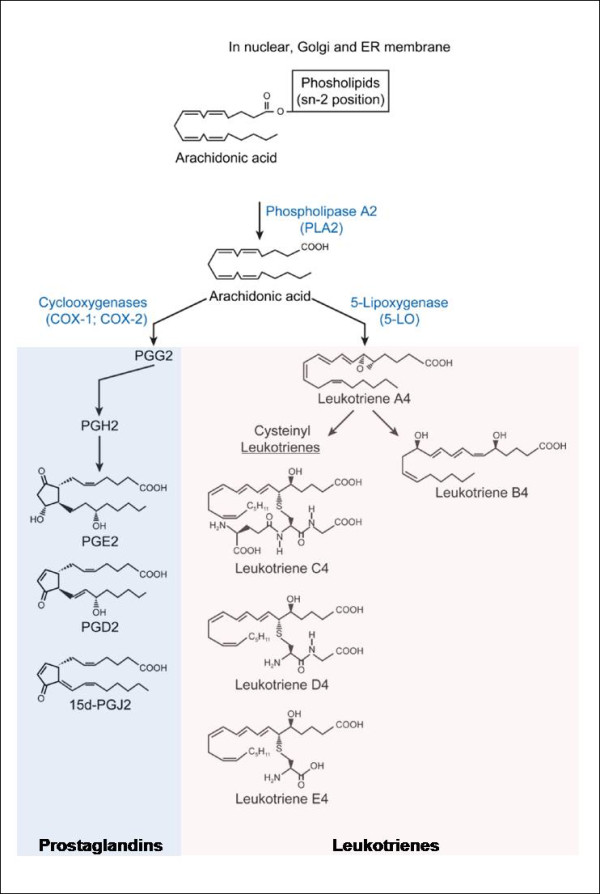
**Metabolic pathway of AA transformation into eicosanoid products**. Once a cell is activated by a proinflammatory stimulus that engages a Gαq-coupled seven-transmembrane receptor, AA can be cleaved by cytosolic PLA_2 _from phospholipids that compose cellular membranes (mostly nuclear, endoplasmic reticulum or Golgi membranes)[173]. Free AA can subsequently be converted into different eicosanoid products by different enzymes (represented in blue), notably the COX and LO pathways. This review will mainly focus on the prostanoids - PGE_2_, and 15d-PGJ_2 _prostaglandins - and on 5-LO products, namely LTs. Only eicosanoids and pathways of interest in this paper are depicted.

Once cells are exposed to different physiological or pathological stimuli such as cytokines, growth factors or physical trauma, AA is cleaved from membrane phospholipids by the action of the enzyme phospholipase A_2 _(PLA_2_) (Figure 1). From then on, the free fatty acid may undergo transformation either through the cyclooxygenase (COX) pathway, giving rise to PGs, or through the lipoxygenase pathway, notably producing LTs. In the latter case, AA is transferred by the five-lipoxygenase activating protein (FLAP) to 5-lipoxygenase (5-LO), an enzyme primarily expressed in most leukocytes [62]. Thereafter, 5-LO converts AA into LTA_4 _[63]. This unstable intermediate can then be metabolized into LTB_4 _by LTA_4 _hydrolase or into LTC_4 _by LTC_4 _synthase [64]. On the other hand, PGs are produced by most nucleated cells in our body, and formed by the rate-limiting COXs. COX-1 is generally thought to be a constitutively expressed enzyme, while COX-2 is highly inducible under inflammatory conditions through the control of the ubiquitous mammalian transcription factor NF-kB [65,66]. COX-2 is especially important in cells that are involved in inflammation, such as macrophages and monocytes, and is responsible for the synthesis of those prostanoids involved in severe inflammatory states. It is noteworthy that, in the brain, an alternatively spliced COX-1 isoform, termed COX-3, might also be in play [67,68]. COX enzymes produce PGH_2_, which is then converted into several isomers via various cell-specific synthases, as reviewed by [69].

### Biological functions

It has been well documented that PGs are important homeostatic or proinflammatory lipid mediators which act in an autocrine and paracrine manner. They are implicated in a wide array of biological functions such as vasoconstriction, vasodilatation, aggregation, chemotaxis, fever, pain response and asthma [70]. This family of potent hormone-like substances include PGE_2_, PGD_2_, and PGF_2α_. As for PGD_2_, it can subsequently be converted into the J class of PGs, which include the cyclopentenone 15d-PGJ_2 _[71-73]. PGE_2 _is a well-known inflammatory modulator implicated in diverse physiological conditions such as vasodilatation, pain, fever, relaxing of smooth muscles, neuronal function and inhibition of noradrenalin release from sympathetic nerve terminals. As for 15d-PGJ_2_, this PG is produced in mast cells, platelets and alveolar macrophages. It has been documented as a potent inflammatory regulator, given its capacity to inhibit cytokine secretion and functions of both antigen-presenting cells and T lymphocytes [74,75].

LTs are potent proinflammatory mediators, which are involved in many innate immunological processes such as leukocyte adhesion, chemotaxis and activation [70,76]. They regulate the immune response to different stimuli, and are known to play an important role in asthma, vascular disease, cancer and other pathological conditions [77]. Produced in leukocytes, LTs include LTB_4 _and several cysteinyl leukotrienes (cysLTs) (e.g. LTC4, LTD4 and LTE4) (Figure 1). LTB_4 _is known as a potent chemoattractant, also capable of inducing the formation of reactive oxygen species and the release of lysosome enzymes by most leukocytes. Known as the slow-reacting substances of anaphylaxis, cysLTs induce inflammation, and may also, when in excess, induce anaphylactic shock. Macrophages produce both LTB_4 _and CysLTs. As part of the innate immune response, antimicrobial effector functions modulated by LTs include direct effects, such as promotion of leukocyte accumulation and enhancement of macrophages and neutrophils microbe phagocytosis and killing capacity, as well as indirect effects, via the production of other inflammatory molecules [78].

The recently-discovered eoxins are closely related to cysLTs, and are also proinflammatory, but are produced via the 12/15-LO pathways. For instance, 15-HETE is produced from AA in most immune cells which carry 15-LO, such as neutrophils [79], endothelial cells [80], and macrophages [81]. This lipid mediator is mainly produced during inflammatory and immunological conditions, and is known to modulate inflammatory diseases by influencing different biological responses such as chemotaxis, hormone secretion, ion transport and various enzymatic pathways in a receptor-specific manner. Although its extracellular receptor remains to be identified, 15-HETE is known to serve as an endogenous ligand for the peroxisome proliferators activated receptors [82,83].

### LT and PG receptors

Eicosanoids mostly exert their effects by binding to seven transmembrane-spanning receptors, more specifically Gq- and Gi-protein coupled receptors (GPCR), found on most leukocytes. Engagement of an LT receptor is generally associated with a decrease of cyclic adenosine monophosphate (cAMP) and an increase in intracellular calcium followed by an activation of phosphatidyl inositol 3-kinase, protein kinase C, MAP kinase, Rac and NF-kB [84-87]. The receptors for LTB_4 _are known as LTB_4 _receptor 1 (LTB_4 _receptor, or BLT1) - a high affinity receptor, and BLT2 - a low affinity receptor. BLT1 is preferentially expressed on leukocytes, while BLT2 is ubiquitously expressed. CysLTs engage cysLT1, cysLT2 or GPR17 receptors [77,88,89], although the latter may regulate the signalling from cysLT1 in response to cysLTs [90].

Regarding PGs, they act on a broader range of receptors. PGE_2_, for example, will engage E-prostanoid receptors EP1, EP2, EP3 or EP4, which each has distinct biochemical properties and tissue and cellular localization, which explains the very disparate effects of this PG, depending on the cell type [91]. As for 15d-PGJ_2_, no specific receptor has been identified to date. Instead, it has been shown to act via PGD_2 _receptors (DP1 and DP2) and through interaction with intracellular targets, including nuclear receptors and non-receptor proteins [92]. In particular, 15d-PGJ_2 _is recognized as the endogenous ligand for the intranuclear receptor peroxisome proliferator-activated receptor-γ (PPAR-γ) [74].

## 4. Known interactions between eicosanoids and HIV, outside the CNS

### PGs and HIV

The anti-HIV properties of PGs are well documented [93-96]. However, only a handful of studies have attempted to elucidate the mechanisms involved. For example, it has been clearly shown that PGE_2 _induces resistance of monocyte-derived macrophages (MDMs) to HIV infection by negatively modulating the surface expression of CCR5 - the main coreceptor used by R5-tropic strains to enter host cells in a pH-independent manner. It does so through augmentation of cAMP cellular concentration [97] which, in turn, activates a protein kinase A (PKA)-dependent mechanism [98]. One may speculate that activation of PKA through a cAMP increase may regulate CCR5 levels by heterologous desensitization (whereby the activation of a GPCR by its agonist causes downregulation of another GPCR on the same cell). In correlation to these findings, a recent study has shown that over-expression of PG synthase-2 greatly reduces the levels of HIV replication in primary human monocuclear cells [99]. These observations are, however, in contrast to a study by Dumais and colleagues showing that PGE_2 _can facilitate HIV production in a T cell line by inducing transcriptional activation of the virus regulatory elements (i.e. long terminal repeat/LTR) [100]. This discrepancy could be explained by the different cell types used in the two studies and the nature of the PGs involved, which do not act via the same signalling pathways. Indeed, PTGS2 is known to down-regulate NF-κB-mediated transcription, whereas PGE2 activates this same transcription factor.

Another potent PG exerting antiviral activities, 15d-PGJ_2_, inhibits transcriptional activation of HIV LTR by antagonizing NF-κB in colonic epithelial cells through a PPAR-γ-independent mechanism [101]. It has been recently observed in human macrophages that 15d-PGJ_2 _can also modify free thiols in the viral transactivating Tat protein, thus suppressing its transactivation function [102]. Altogether, certain PGs can now be considered as anti-HIV eicosanoids and may very well contribute to the limitation of the systemic viral load in HIV-infected individuals.

### LTs and HIV

Several studies have already shown how endogenous LTs play a critical role in the innate immune defence against bacterial and viral infections [103-107]. Regarding the response to HIV, most of our knowledge concerns LTB_4_. As early as 1989, Thorsen and colleagues noticed that neutrophils isolated from patients infected with HIV display a reduced capacity of producing LTB_4 _[108]. Another group later demonstrated that, in HIV-infected subjects, a defect could be observed in the 5-LO metabolic capacity of lung alveolar macrophages, which are resident cells primarily responsible for maintaining the sterility in the lungs. This diminished 5-LO efficiency was associated with a decreased expression of both 5-LO and FLAP in HIV-infected patients displaying low CD4 T-cell counts (< 200/mm^3^) [109]. The same tendencies were observed in polymorphonuclear cells (PMNs) from subjects with low CD4 T-cell counts [110]. This decrease of LT synthesis capacity in alveolar macrophages and PMNs may negatively modulate the innate immune response to challenges by pathogens and opportunistic infections in the lungs during the late phase of HIV infection. For example, 5-LO knockout (KO) mice have a reduced survival rate after being inoculated intratracheally with *K. pneumoniae*, as compared to wild-type mice [111].

Recently, the existence of several 5-LO isoforms has been discovered in different human cell types (i.e. monocytes, B cells and PMNs). All cells examined express the full-length mRNA. On the other hand, the alternative forms of 5-LO appear to be expressed in a cell-type or cell-line specific manner. By themselves, these alternative isoforms are enzymatically inactive. Interestingly, the coexpression of these isoforms with the full-length 5-LO affects the capacity of cells to synthesize LTs [112]. How HIV infection can negatively modulate the LT production capacity of cells (i.e. alveolar macrophages and PMNs) remains to be explained. One may speculate that the 5-LO inefficacy brought about by HIV infection might be due to the upregulation of inactive 5-LO isoforms in cells, resulting in a downmodulation of their LT synthesis capacity. Additional studies are warranted to validate this hypothesis.

In general, LTs are considered to be mediators of antimicrobial host defence. In humans, administration of LTB_4 _to healthy individuals induces a plasmatic increase of α-defensins and MIP-1β, both well-known antiviral proteins. Interestingly, PMNs isolated from HIV-infected and non-infected subjects secrete similar amounts of α-defensins in response to LTB_4 _activation [113], meaning that this LT can induce an antiviral response whether or not PMNs are exposed to HIV. Moreover, the secretome of PMNs obtained from healthy individuals treated with LTB_4 _contains several antimicrobial proteins including α-defensins, cathepsin G, elastase, lysozyme C, and LL-37, which significantly attenuates infectivity of both X4- and R5-tropic HIV variants [114].

### Non-steroidal anti-inflammatory drugs and HIV

As they constitute COX inhibitors, it is relevant to discuss here the anti-HIV potential of non-steroidal anti-inflammatory drugs (NSAIDs). In the HAART era, rheumatic manifestations have been reported to affect 9% of HIV-infected patients [115], and up to 60% of untreated individuals [116-118]. In such cases, NSAIDs are often the first line of medication prescribed. Indeed, many anti-inflammatory compounds (such as platelet-activating factor (PAF) receptor antagonist, aspirin and indomethacin) have demonstrated a certain anti-HIV potential. Interestingly, o-(acetoxyphenyl)hept-2-ynyl sulfide (APHS), an aspirin-derived molecule, has proven to be a compound with important anti-HIV properties. More precisely, APHS can act as a potent HIV inhibitor by negatively modulating Gag DNA synthesis during the reverse transcription (RT) process [119]. In addition, APHS can also act synergically with clinically available reverse transcriptase and protease inhibitors to inhibit several strains of HIV under *in vitro *conditions [120]. However, not all NSAIDs show anti-HIV properties. For instance, we have evaluated whether NS-398, another potent COX inhibitor [121], albeit specific for COX-2, could affect HIV replication in both MDMs and MDMi. We found that NS-398 does not significantly modulate HIV infection in these two distinct cellular models (unpublished data). This indicates that some mechanism other than the anti-COX-2 activities of APHS must be responsible for its anti-HIV potential. Interestingly, a recent clinical study has shown that celecoxib, another widely used COX-2 inhibitor, when administered to HIV-infected patients without antiretroviral treatment, downmodulates the immune activation related to clinical progression of chronic HIV infection and improves T cell-dependent functions *in vivo*. Indeed, a reduction in surface expression of CD38 and programmed death-1 is observed in cytotoxic T cells. Furthermore, the celecoxib treatment enhances the number of regulatory T cells and improves the humoral memory recall responses to a T cell-dependent vaccine [122]. Given the obvious beneficial potential of NSAIDs ability to modulate, either directly or indirectly, the replication cycle of HIV and the progressive immune dysfunctions occurring throughout the course of the disease, a better characterization of the mechanisms of action of these drugs is warranted.

## 5. Eicosanoids present in the CNS

Eicosanoids produced in the CNS play an important role in neuropathogenesis. Several different studies, mainly based on murine models, have shown that microglial cells produce many COX and 5-LO products, namely PGE_2_, PGD_2_, LTB_4_, LTC_4_, 5-HETE, 11-HETE and 15-HETE [123-128]. Brain eicosanoids may also be secreted by cell types other than microglia, such as astrocytes, oligodendrocytes, neurons and endothelial cells, which play an important role in the propagation and maintenance of neuroinflammation [129,130].

Not surprisingly, eicosanoid-producing enzymes COXs and lipoxygenases have also been detected in the CNS, either at the mRNA or protein level (Table 1). These enzymes are known to be stimulated during neuroinflammation [131]. For example, 5-LO is expressed in the CNS and has been known to be present in neurons, microglia and astrocyte cells [126,132-136]. As for COX-2, it is induced in most cells of the CNS, including neurons, microglia, astrocytes and oligodendrocytes [128,137-139]. Furthermore, microarray analysis has revealed that activation of mixed glial cells (predominately microglia) leads to striking increases in the expression of FLAP, 5-LO, 15-LO, COX-2, and PLA_2_, which are all HAD-associated gene products involved in LT or PG synthesis [27]. Moreover, a COX-1 splice variant, termed COX-3 or COX-1b, has also been detected at both the mRNA and protein levels in human brain tissues [140]. It has been shown to catalyze the synthesis of PGF_2□_, although less efficiently than COX-1.

**Table 1:** Summary table of the different receptors and eicosanoid-producing enzymes known to be found in the CNS and discussed in this review.

Cells	Receptors	Enzymes
Microglia	cysLT1 ^131^	COX-2 ^123, 127, 135, 138^
	cysLT2 ^124^	5-LO ^124, 125, 132^
	EP4 ^141^	FLAP ^132^
	EP2 ^141^	COX-1 ^128, 135, 137^
		PLA2 ^27^

Astrocytes	EP2 ^141^	5-LO ^133^
		COX-2 ^138^
		PLA2 ^129^
		12-LO ^130^

Oligodendrocytes	GPR17 ^145^	COX-2 ^138^
		12-LO ^130^

Neurons	BLT1 ^131^	5-LO ^133, 135^
	GPR17 ^89, 145^	COX-2 ^128, 134^
	cysLT2 ^144^	12-LO ^130^
	EP2 ^141^	
	EP1 ^141^	
	EP3 ^141^	
	EP4 ^141^	

Endothelial cells	cysLT2 ^147^	
	EP4 ^141^	

Unspecified	DP1-2^128^	
CNS cell types	FP^128^	
	IP^128^	

Different prostanoid receptors, such as EP1-4 (receptor for PGE_2_), DP1-2 (receptor for PGD_2_), FP (receptor for PGF_2α_), and IP (receptor for PGI_2_), are found in the CNS, and their level of expression is often linked to the severity of cognitive impairment [128,141,142] (Table 1). Meanwhile, the main LT receptors expressed in the human and murine CNS, more specifically by neurons, endothelial and glial cells, are cysLT receptors, mostly cysLT2 and GPR17 [89,143-148].

The eicosanoids present in the CNS, whether or not they are produced by microglia, contribute greatly to neuroinflammation and HAD, as proinflammatory lipid mediators. In fact, the expression profile of 5-LO and COX products and of their receptors might serve as biomarkers for detecting CNS pathologies and their degree of severity.

## 6. Interactions between HIV and eicosanoids in the CNS

It is well known that elevated amounts of eicosanoids can be found in the brain and CSF of HIV-infected individuals [142,149]. Likewise, an upregulation of COX-2 expression is observed in *in vitro *cocultures of HIV-infected macrophages and brain endothelial cells, as well as in brain-derived cell lines exposed to soluble viral proteins [150-152]. We are, however, only starting to understand how eicosanoids interact with HIV. This section will focus on the complex direct or indirect interactions between HIV and COX-2, 5-LO and their products in the CNS, and on how this interplay might influence the progression of HIV-associated neurological impairments (Table 2).

**Table 2:** Summary table of the known relationships between eicosanoids and HIV in the CNS.

Exposure to	Type of cells or mode of exposure	Species	Product	Effect	Mediated by	Citation
gp120	intracerebral injection	rat	PGE2	neuronal apoptosis	IL-1β	159, 160

gp120	intracerebral injection	rat	PGE2	neuronal apoptosis	*N/A*	161

gp120	astrocytes	human	PGE2	neurotoxicity	*N/A*	162

gp120	neurons	human	*N/A*	neurotoxicity	5-LO/PGHS	172

Tat	intracerebral injection	mouse	COX-2	Neuroinflammation	NFKb	166

Tat	tail vein injection	mouse	COX-2	BBB disruption	*N/A*	165

LTs	microglia	human	*N/A*	HIV inhibition	PKC	unpublished

HIV-1	endothelial cells/macrophages	human	COX-2	Neuroinflammation	IL-1b	150

N/A: Non available data

PGHS: prostaglandin H synthase

### Relationship between HIV and COX-2 and its products

Although it is widely accepted that neurons do not support productive infection with HIV, there is clear evidence of neuronal loss in brains of HIV-infected individuals [44,153,154]. The viral surface glycoprotein gp120 is known to be greatly responsible for inducing neuroinflammation and neurodegeneration [155-158]. Interestingly, *in vitro *and *in vivo *studies have shown that enhanced COX-2 expression and PGE_2 _synthesis are involved in apoptosis observed in the CNS of rats following intracerebroventricular injection of gp120 [159-161]. Moreover, the varying degree of severity of neuroinflammation brought about by gp120 from different HIV clades is notably linked to the level of synthesis of certain COX products. Indeed, a recent study has indicated that human primary astrocytes exposed to purified gp120 from HIV clade B produce higher levels of COX-2 than astrocytes exposed to gp120 from clade C [162]. However, the mechanisms through which gp120 induces COX-2 expression in the brain are still not fully understood. *In vitro *and *in vivo *data have revealed a role for IL-1β in mediating neurotoxicity induced by gp120 [163,164]. It has been shown that HIV gp120 can induce COX-2 expression not only in neuroblastoma cells, but also in astrocytoma cells through an NF-κB-mediated signal transduction pathway [151,152]. Furthermore, a recent study shows that the capacity of gp120 to induce COX-2 and its products in microglial cells greatly depends on the presence of IFN_γ _[7]. These data suggest that the IL-1β and IFN_γ_-mediated COX-2 expression and PGE_2 _synthesis triggered by HIV gp120 in the CNS may account for the observed neuronal dysfunction. Thus, gp120 may be considered as a viral factor that potently induces neuroinflammation via COX-2 by interacting directly with cells of the CNS.

Another viral protein known to be released in the surrounding environment following HIV infection is Tat. It has been shown that both Tat and COX-2 are involved in the neuropathogenesis associated with HIV infection. For example, the upregulated expression of COX-2 in the BBB endothelial cells and macrophages brought on by HIV infection has been attributed, at least partly, to Tat-induced alterations of occludin expression, leading to the loss of tight junction integrity and the BBB breakdown [150,165]. Furthermore, an increase in COX-2 expression and PGE_2 _production is observed in astrocytoma cell lines and primary human astrocytes treated with Tat [166]. The intrahippocampal injection of Tat in mice induces a COX-2 expression not only in astrocytes, but also in microglial cells. Moreover, the proinflammatory effect of Tat is significantly attenuated upon treatment with the COX-2 inhibitor NS-398, which demonstrates the involvement of COX-2 in the Tat-induced neuropathogenesis [167]. Hence, exposure of astrocytes and microglia to either fully competent HIV particles or viral proteins promotes secretion of several proinflammatory molecules, including eicosanoid products, which greatly influences neuroinflammation and contributes to the severity of HIV-associated neurocognitive complications. This may be further explained by recent findings indicating that coculture with HIV-infected astrocytes is sufficient to increase the production of neurotoxic molecules released by virus-infected microglia such as PPP4R2, HSPA9 and NAP1L1 (proteins that regulate neuronal apoptosis) [61]. Although the role of COX-2 in brain inflammation is still not fully recognized, one may speculate that neurological complications that occur throughout the course of HIV infection may be in part mediated by COX-2 and its products. Another point to consider concerning HIV-infected patients is the potential effect of antiretrovirals on eicosanoids. For instance, maraviroc, a widely used CCR5 antagonist, is known to induce PG synthesis in microglia, thus strengthening their proinflammatory activation, which is likely to exacerbate neurological complications [7].

### Relationship between HIV and LTs

Although previous studies have produced evidence of the anti-microbial properties of LTs, only recently have we established the relationship between LTs and viral infections in the CNS. For example, Chen and colleagues have shown that, during early infection with vesicular stomatitis virus, mice treated with the 5-LO antagonist Zileuton and 5-LO knock-out mice both presented an impaired infiltration process of neutrophils into the CNS, as well as fewer neurons expressing nitric oxide synthase-1, higher viral titres and increased disruption of the BBB [104]. These data clearly showed the protective role of LTs during viral infections in the CNS.

We now know that LTs are present in the CNS of HIV-infected individuals, where they can be produced by different cell types, including monocytes and microglia [125,126,149,168]. Moreover, recent studies have shown that high concentrations of eicosanoids, including LTB_4_, and several other AA cascade markers are found in the brain of HIV-transgenic rats [169,170]. In fact, cocultures of astroglial cells and HIV-infected monocytes release high levels of AA-derived metabolites, such as LTB_4 _and LTD_4 _[171], which can lead to neuroinflammation and neurotoxicity. Furthermore, gp120 induces necrotic death of human neuroblastoma cells by activating 5-LO [172]. This issue is, however, not without controversy since it has been shown that expression and activity of 5-LO can actually decrease in the rat neocortex upon the intracerebroventricular injection of gp120 [161]. The vast differences between the microenvironment of neuronal cells found in the rat brain neocortex upon gp120 intrathecal injection and the one studied in an *in vitro *experimental setup consisting of a single human cell type may account for the observed opposite effects of gp120 on 5-LO regulation.

Interestingly, our laboratory has shown that LTB_4 _and LTC_4 _negatively modulate HIV infection in MDMi's in a protein kinase C-dependent manner (unpublished observations). This phenomenon may be explained in part by the fact that LTs inhibit the pH-independent fusion step of the infection by decreasing the levels of CCR5 on the cell surface. Moreover, decreased amounts of proviral DNA are found in LT-treated MDMi's, while RT products remain unaffected by LTs, indicating a blockage between the RT and integration steps of the infection.

Although LTs present in the CNS may exert an anti-HIV effect, it is possible that HIV can counteract this phenomenon by inhibiting their production. On the other hand, a neurotoxic response induced by HIV infection in the CNS can also be mediated through 5-LO products. The balance between the anti-HIV effect of LTs and their neuroinflammatory/neurotoxic effect might be pivotal in the development of neuroAIDS. Further research will be needed to better define the role played by LTs in the neuropathogenesis associated with HIV infection.

## 7. Conclusion

In summary, HIV-associated neurological impairments continue to be an important issue in the HAART era. We have learned that HIV-induced inflammation in the CNS, caused primarily by microglia and astrocytes, is greatly responsible for the neurological symptoms observed in virus-infected individuals. Importantly, multiple markers of brain AA metabolism are upregulated in the HIV-infected CNS, which may be correlated with dementia severity and behavioural deficits. The direct role of these potent inflammatory lipid mediators in the development and progression of the disease is still unfortunately poorly understood. Recent advances in the field have shown that COX and 5-LO products can negatively modulate HIV infection in different host cells. Therefore, PGs and LTs produced by innate immune cells may have an important role in controlling HIV replication, maybe more specifically in the CNS. This may explain, at least partially, how the viral load is often maintained at lower titres in this compartment than in the peripheral blood; the degree of penetration of antiretroviral agents in the CNS and their efficacy in the brain tissue also remain, of course, to be fully investigated.

We should examine more closely eicosanoid products in the CNS of infected individuals in the perspective of their ability to control the viral burden in this compartment, even though they are often associated with neuroinflammatory complications. In other regards, it would be worth evaluating the usefulness of eicosanoids as biomarkers to assess the severity of HIV-induced neurological impairments.

## Competing interests

The authors declare that they have no competing interests.

## Authors' contributions

JB carried out most of the literature research and the first draft. SM participated in the drafting and research of the literature. CB and MJT carefully reviewed the manuscript. All authors read and approved the final manuscript.
